# Cadaveric anatomy and dissection in surgical training

**DOI:** 10.4274/tjod.galenos.2018.15931

**Published:** 2019-03-27

**Authors:** İlker Selcuk, Ilkan Tatar, Emre Huri

**Affiliations:** 1 University of Health Sciences, Zekai Tahir Burak Woman’s Health Training and Research Hospital, Clinic of Gynecologic Oncology, Ankara, Turkey; 2 Hacettepe University Faculty of Medicine, Department of Anatomy, Ankara, Turkey; 3 Hacettepe University Faculty of Medicine, Department of Urology, Ankara, Turkey

**Keywords:** Anatomy, surgery, education, cadaver, gynecology

## Abstract

Detailed knowledge of anatomy is an essential part of surgical practice. However, there are many drawbacks in anatomy education that make many residents feel inadequate when they start performing surgeries. Cadaveric dissection courses aim to close the gap between the anatomic knowledge and surgical practice. This review focuses on the role of cadaveric dissection on surgical education, and additionally states the panel decision of the Surgical Anatomy and Technologies Association on the proper use of cadavers.

## Introduction

This review covers the concept of cadaveric dissection in terms of teaching anatomy and post-graduate surgical training.

### The role of cadaveric dissection in teaching anatomy

Cadaveric dissection has been the main teaching modality in anatomy education since the ancient times. In the 3^rd^ century before Christ, the first human cadaveric dissections were performed in Greece by Herophilus of Chalcedon and Erasistratus of Chios to understand the whole body from the viewpoint of anatomy and physiology. However, religious and moral attitudes and taboos towards physicians and medical schools had many detrimental effects on the scientific value of cadaver-based education^(1)^.

Cadaveric dissection is the traditional way of teaching anatomy after theoretical lessons and discussions on the atlas images^(2)^. Medical students gain knowledge and strengthen theoretical data through visualization of real anatomic structures. Additionally, by practicing on cadavers they touch and feel the anatomic relations more efficiently^(3)^. Owing to the role of cadaveric dissection in generating a three-dimensional (3D) perspective, and providing an easy way of understanding and recalling anatomic structures, the literature indicates that cadaveric dissection is one of the most powerful ways of teaching topographic and regional anatomy^(1,4)^.

Recently, there has been a trend shift in modern anatomy education, and many novel options are used in teaching anatomy. Cadaveric dissection and didactic lessons with atlas images constitute the traditional methods; however, 3D simulation technologies, virtual/augmented reality, 3D printed materials, simulation/training models, and radiology-based comparative illustrations form the new methodologies in current anatomy education^(5)^.

### Cadaveric dissection and post-graduate surgical education

Post-graduate anatomy education is the core praxis in improving surgical and technical knowledge for surgery residents. Simulation-based education with hands-on courses and cadaveric dissections focus on a detailed practice of surgical procedures prior to live patient operations, consequently an increase in confidence levels and surgical skills of residents will be noticed^(6)^. Nevertheless, cadaveric dissection is not the sole method of teaching anatomy and it should be complemented by other innovative educational tools. In this way, medical students and residents will be better equipped and well prepared for future medical activities^(7)^.

The problem beyond the inadequacy in anatomy education for surgery residents gave rise to the wide use of cadaveric courses in many surgical disciplines. Giving the opportunity to residents to attend these cadaveric courses will improve the educational quality during the first steps of learning surgical procedures by raising self-confidence and better surgical skills^(8)^.

### Cadaveric courses in obstetrics-gynecology and other surgical disciplines

Cadaveric dissection under the supervision of senior surgeons and anatomists will develop a practical competency in the field of minimally invasive surgery, gynecology, gynecologic oncology, and urogynecology, even if in obstetrics, and also for the management of complications^(9)^.

Sharma et al.^(10)^ reported the positive effect of procedure-oriented cadaveric courses, which target teaching specific operations to improve operative confidence and surgical technique. Some advanced surgical procedures could be observed or performed for the first time at the cadaveric courses, which deeply enhance anatomic knowledge and may also provide a higher level of autonomy to perform procedures independently^(6)^.

Post-course surveys showed that improvements in surgical skills, which are transferrable to the operating room, were essentially maintained by cadaveric dissections^(11)^ and Lim et al.^(8) ^showed that the best improvements were observed in hysterectomy and salpingo-oophorectomy procedures for gynecology practice. Both basic and advanced surgical procedures can be performed on cadavers and the use of fresh frozen cadavers instead of traditional embalmed ones make it easy to learn the general objectives of a surgical operation in which a special and tailored practice is needed^(12)^. Simulation of surgical scenarios, identifying the steps of new techniques, and teaching the tips and tricks of advanced surgical procedures are effectively performed by using fresh frozen cadavers for postgraduate surgical education.

Previously, a newer surgical method used in urogynecology practice, transobturator tape procedure, was widely discussed in cadaveric illustration studies^(13,14)^. Safe implementation of surgical procedures with regard to vascular and neural structures was also studied for transvaginal tape and sacrospinous ligament fixation operations in urogynecology literature^(15,16)^.

Additionally, the complex structure of pelvic anatomy and the pathway of the ureter were also demonstrated in cadaveric studies for surgical education^(17,18,19)^. Soft-preserved cadavers, which are prepared using phenol, alcohol, and glycerol, were used by Barton et al.^(20)^ during a gynecologic oncology cadaveric course, and the need of such a cadaveric anatomy education during gynecologic oncology fellowship was the objective result of the end-course evaluation.

In addition, management of complications is a major requirement that a resident needs to learn during the surgical training period; however, it is not easy to observe these kinds of cases all the time. Cadaveric courses can mimic complications through simulations and this yields a higher practice level and competency for less common procedures and real-life conditions^(21)^.

In practical obstetrics, cadaveric education increased the ability to manage surgical cases where a serious episiotomy tear or a massive peripartum bleeding occurred^(22,23,24)^.

Minimally invasive gynecology, with the advantage of video recording, especially in the laparoscopic technique, is a well-known surgical practice to popularize the surgical approach and maneuvers to a widespread population, and this is also valid for teaching and demonstrating anatomic structures. Cundiff et al.^(25)^ evaluated the effectiveness of laparoscopic cadaveric dissection after starting the residency program of obstetrics and gynecology to overcome the deficiencies in anatomy education; the course produced satisfactory results for the participants and also allowed video recording, which could spread the educational materials to non-active participants.

Other surgical branches also benefit from cadaveric courses; residents, fellows, junior surgeons, and new consultants gain experience and improve skills with confidence. Cadaveric courses are also the approved method of surgical education to share knowledge in brain surgery, plastic surgery, orthopedics, general surgery, urology, vascular surgery, and other branches. Cadaveric courses will lead to improvement of surgical outcomes by means of identifying proper anatomical landmarks, practicing more without any stress of the operation room in a comfortable environment, and teaching the basis of a new modification in a surgical approach^(26,27,28,29,30)^.

### Panel decision on the “Ethical and Proper Use of Cadavers in Surgical Education”

Surgical Anatomy and Technologies Association (SATA) organized a panel titled “Ethical and Proper Use of Cadavers in Surgical Education” during the First National Anatomy and Cadaveric Dissection Symposium of SATA, which was held at Kars, Turkey, in March 2018. More than 50 participants attended this interactive session, which was conducted by three mentors (EH, IT, IS) who were proficient in surgery and anatomy. The role of cadaveric dissection in surgical education was discussed in many aspects.

The final decisions on the Ethical and Proper Use of Cadavers in Surgical Education:

-The scientific committee of cadaveric courses should be consisted of phyicians and anatomists, a multidisciplinary/advisory board.

-Cadaveric courses without the integration of anatomy departments and anatomists will give harm to an effective education.

-Dissection subgroups will improve the surgical education both for mentors and residents.

-A certification program should be formed to train the teachers of cadaveric surgical education (“teach the teachers”).

-Number of participants per cadaver during the cadaveric dissection courses should be defined according to the aim and tasks of the course to maintain a cost-effective and educational activity.

-Criteria of a well-designed cadaveric course should be established with respect to the training aim, ethics, and health conditions of the environment.

-Cadaveric dissection laboratories should be high quality to preserve the health conditions of the participants and laboratory personnel.

-A rotation program to anatomy departments should be implemented to surgical residency education to improve anatomic knowledge.

Despite the higher costs of cadavers, if used effectively and properly, by conducting small but meticulous dissections, cadaveric courses will produce successful educational results in a cost-effective manner. However, measurements of the validity and effectiveness of these gains are lacking during surgical operations, as such there is a need for further studies in this area^(31)^.

## Conclusion

In conclusion, to gain surgical skills, experience, and confidence before performing a procedure on a live patient, hands-on cadaveric courses should be an integrated part of surgical education during residency and the post-graduate period. Fundamentally, these courses should be planned under the supervision of an advisory board consisting of both surgeons and anatomists with limited numbers of participants to allow each attendee achieve the final goals of the course.
